# Identification of natural compounds targeting Annexin A2 with an anti-cancer effect

**DOI:** 10.1007/s13238-018-0513-z

**Published:** 2018-03-05

**Authors:** Yu-Shi Wang, He Li, Yang Li, Hongyan Zhu, Ying-Hua Jin

**Affiliations:** Key Laboratory for Molecular Enzymology and Engineering of the Ministry of Education, College of Life Science, Jilin University, Changchun, 130012 China

**Keywords:** Annexin A2, G-Rg5, G-Rk1, HCC, NF-κB

## Abstract

Annexin A2, a multifunctional tumor associated protein, promotes nuclear factor-kappa B (NF-κB) activation by interacting with NF-κB p50 subunit and facilitating its nuclear translocation. Here we demonstrated that two ginsenosides Rg5 (G-Rg5) and Rk1 (G-Rk1), with similar structure, directly bound to Annexin A2 by molecular docking and cellular thermal shift assay. Both Rg5 and Rk1 inhibited the interaction between Annexin A2 and NF-κB p50 subunit, their translocation to nuclear and NF-κB activation. Inhibition of NF-κB by these two ginsenosides decreased the expression of inhibitor of apoptosis proteins (IAPs), leading to caspase activation and apoptosis. Over expression of K302A Annexin A2, a mutant version of Annexin A2, which fails to interact with G-Rg5 and G-Rk1, effectively reduced the NF-κB inhibitory effect and apoptosis induced by G-Rg5 and G-Rk1. In addition, the knockdown of Annexin A2 largely enhanced NF-κB activation and apoptosis induced by the two molecules, indicating that the effects of G-Rg5 and G-Rk1 on NF-κB were mainly mediated by Annexin A2. Taken together, this study for the first time demonstrated that G-Rg5 and G-Rk1 inhibit tumor cell growth by targeting Annexin A2 and NF-κB pathway, and G-Rg5 and G-Rk1 might be promising natural compounds for targeted cancer therapy.

**Electronic supplementary material:**

The online version of this article (10.1007/s13238-018-0513-z) contains supplementary material, which is available to authorized users.

## Introduction

Annexin A2, a Ca^2+^-dependent lipid binding protein, widely distributed in nuclear, cytoplasm, endosomes and extracellular space and participates in various cellular process including signal transduction, endocytosis and exocytosis, proliferation, differentiation and apoptosis in mammalian cells (Klionsky et al., 2012; Moreau et al., 2011; Gerke et al., 2005; Moss and Morgan, 2004). A lot of evidence suggests that Annexin A2 may be a promising therapeutic target for cancer treatment (Chen et al., 2015; Staquicini et al., 2017; Kesavan et al., 2010; Wang et al., 2012). First, an accumulating observations have shown that Annexin A2 overexpressed in various types of cancer cells such as hepatocellular carcinoma (Zhang et al., 2012; Longerich et al., 2011; Sun et al., 2013), breast cancer (Deng et al., 2013; Chuthapisith et al., 2009; Shetty et al., 2012), lung cancer (Yao et al., 2009), gastric carcinoma (Zhang et al., 2012), pancreatic cancer (Takano et al., 2008), and colorectal cancer (Yang et al., 2013; Duncan et al., 2008). Second, the abnormal up-regulation of Annexin A2 enhances cancer development with higher aggression and poorer prognosis (Ohno et al., 2009; Ma et al., 2014; Yang et al., 2014). Third, forced inhibition of Annexin A2 effectively reduces tumor progression both *in vitro* and *in vivo* studies. Furthermore, signal transduction study has shown that Annexin A2 either promotes cancer cell metastasis by forming heterotetramer with S100A10 and binding with cell membrane (Réty et al., 1999), or largely enhances pro-survival capability and chemoresistance by activating transcription factors STAT-3 and nuclear factor-kappa B (NF-κB) (Tong et al., 2015; Jung et al., 2015).

NF-κB is a transcription factor activating the expression of genes involved in inflammation, immune response, angiogenesis, cell proliferation and apoptosis (Perkins, 2012). Constitutive activation of NF-κB is a hallmark of several types of cancers, which not only promotes tumorigenesis and cancer development but also enhances drug resistance (Dolcet et al., 2005; Hanahan and Weinberg, 2000). Recent reports have shown that inhibition of NF-κB significantly inhibited cancer growth by directly inducing cancer cell apoptosis (Woo et al., 2016; Alexandersavino et al., 2016; Kwon et al., 2016; Yin et al., 2016; Hayden et al., 2008). Interestingly, the multifunctional tumor associated protein Annexin A2 promotes NF-κB activation via directly binding to NF-κB p50 subunit with its N-terminal sequences, and inhibition of Annexin A2 provides a new regulatory tool on NF-κB activity (Jung et al., 2015).

Ginsenosides are the major bio-active component of ginseng (Attele et al., 1999) and ginsenoside Rg5 (G-Rg5) and Rk1 (G-Rk1) are the two main ginsenosides identified in heat processed ginseng (Toh et al., 2010). In the present study, we identified G-Rg5 and G-Rk1 that inhibited NF-κB activation, down-regulated the expression of IAPs and induced apoptosis by specifically binding to Annexin A2.

## Results

### G-Rg5 and G-Rk1 interacted with Annexin A2

**Figure 1:**
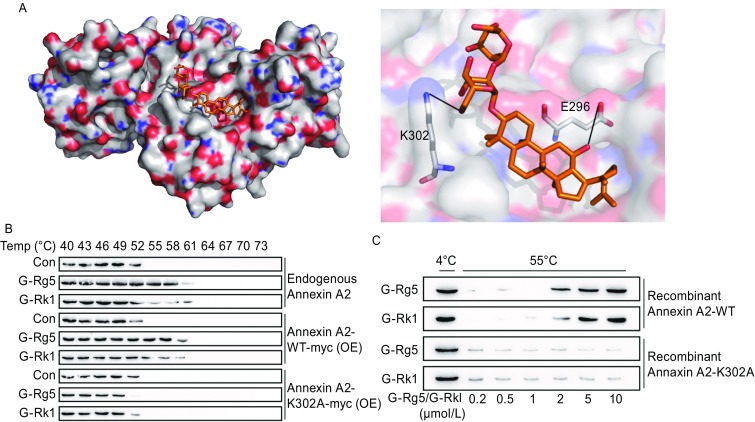
**G-Rg5 and G-Rk1 bound to Annexin A2**. (A) Results of molecular docking shows the interaction between Annexin A2 and G-Rg5. (B) Cellular thermal shift assay presented the thermal stability of Annexin A2 under treatment with G-Rg5 (12 μmol/L) and G-Rk1 (12 μmol/L). (C) Thermal shift *in vitro* showed the thermal stability of purified Annexin A2 (0.2 μmol/L) under treatment with G-Rg5 and G-Rk1

Molecular docking was first performed as a primary screening for natural compounds targeting Annexin A2. G-Rg5 bound to Annexin A2 with Glu296 and Lys302 responsible to this interaction (Fig. 1A). Cellular thermal shift assay (CTSA) with HepG2 cells demonstrated that G-Rg5 and G-Rk1 largely improved the thermal stability of Annexin A2, indicating the binding of G-Rg5 and G-Rk1 with Annexin. Exogenous wild-type Annexin A2 (Annexin A2-WT) showed a similar thermal stability shift under treatment of G-Rg5 and G-Rk1, while G-Rg5 and G-Rk1 didn’t change the thermal stability of exogenous K302A mutant of Annexin A2 (Annexin A2-K302A), indicating Lys302 might be responsible for the interaction between Annexin A2 and ginsenosides (Fig. 1B), as predicted by molecular docking. To rule out the effects from other cellular content and signaling towards the thermal stability of Annexin A2, we carried out the thermal shift assay with purified Annexin A2 and the two ginsenosides. Both G-Rg5 and G-Rk1 obviously enhanced the thermal stability of Annexin A2-WT but not Annexin A2-K302A in a dose-dependent manner (Fig. 1C). These data indicated that both G-Rg5 and G-Rk1 directly bound to Annexin A2, and Lys302 is a critical residue for this interaction.

### G-Rg5 and G-Rk1 inhibited the interaction and nuclear co-localization of Annexin A2 and NF-κB p50 subunit

**Figure 2:**
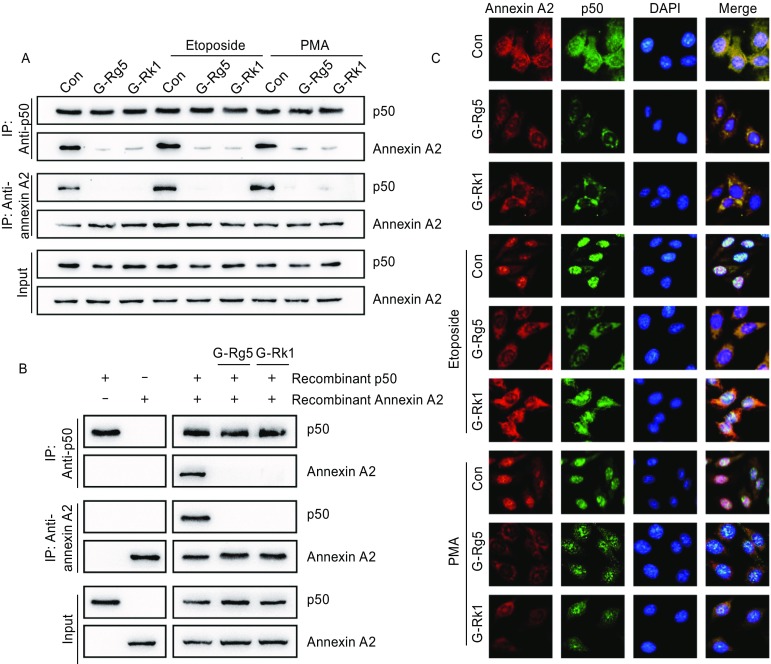
**G-Rg5 and G-Rk1 inhibited interaction between Annexin A2 and NF-κB p50 subunit and their nuclear co-localization**. (A) Immunoprecipitation was performed with whole-cell lysate of HepG2 cells under treatment with G-Rg5 (6 μmol/L), G-Rk1 (6 μmol/L), etoposide (25 μg/mL) and PMA (100 ng/mL), and the interaction was analyzed by an immunoblot. (B) Immunoprecipitation was performed with prokaryotic cells-expressed Annexin A2 and NF-κB p50 subunit under treatment with G-Rg5 (6 μmol/L) and G-Rk1 (6 μmol/L), and the interaction was analyzed by an immunoblot. (C) The subcellular distribution of Annexin A2 and NF-κB p50 subunit was examined by immunofluorescence under treatment with G-Rg5 (6 μmol/L), G-Rk1 (6 μmol/L), etoposide (25 μg/mL) and PMA (100 ng/mL), and DAPI showed the nuclear region

In order to tell whether the G-Rg5 and G-Rk1 binding to Annexin A2 modulated the action of Annexin A2 in cells, we determined the interaction and nuclear co-localization of Annexin A2 and NF-κB p50 subunit under G-Rg5 and G-Rk1 treatment. Immuno-precipitation was performed using HepG2 cells treated with 6 μmol/L G-Rg5 or 6 μmol/L G-Rk1, with or without NF-κB activator PMA (100 ng/mL) or etoposide (25 μg/mL). The interaction between Annexin A2 and NF-κB p50 was significantly attenuated under G-Rg5 and G-Rk1 treatment, both in resting cells and NF-κB activator treated cells (Fig. 2A). To rule out the possibility that the interaction was regulated by other eukaryotic cellular content, another immunoprecipitation was carried out with prokaryotic cell-expressed Annexin A2 and p50, and the interaction was also inhibited largely by G-Rg5 and G-Rk1 (Fig. 2B). Next, immunofluorescence was employed to examine the sub-cellular distribution of Annexin A2 and p50 under ginsenoside treatment. The nuclear co-localization of Annexin A2 and p50 was inhibited in both resting state and NF-κB activator-stimulating cells under G-Rg5 and G-Rk1 treatment (Fig. 2C). Taken together, G-Rg5 and G-Rk1 inhibited the interaction between Annexin A2 and p50, and their nuclear co-localization.

### G-Rg5 and G-Rk1 inhibited NF-κB activation and down-regulated downstream anti-apoptosis gene expression

**Figure 3:**
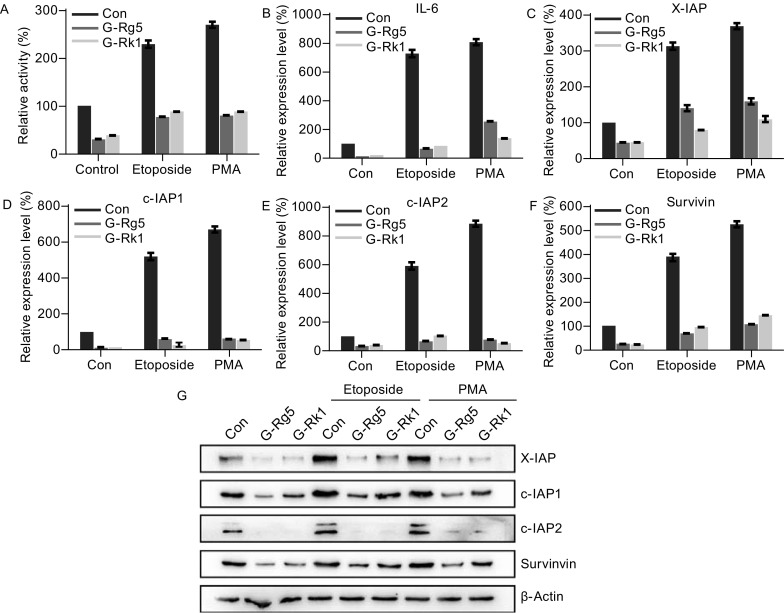
**G-Rg5 and G-Rk1 inhibited NF-κB activation and down-regulated downstream anti-apoptosis genes**. (A) NF-κB activity was examined by luciferase reporter assay under treatment with G-Rg5 (6 μmol/L), G-Rk1 (6 μmol/L), etoposide (25 μg/mL) and PMA (100 ng/mL). (B–F) Relative gene expression levels of IL-6 (B), X-IAP (C), c-IAP1 (D), c-IAP2 (E) and survivin (F) were examined by qRT-PCR under treatment with G-Rg5 (6 μmol/L), G-Rk1 (6 μmol/L), etoposide (25 μg/mL) and PMA (100 ng/mL). (G) Protein levels of X-IAP, c-IAP1, c-IAP2 and survivin were examined by immunoblot under treatment with G-Rg5 (6 μmol/L), G-Rk1 (6 μmol/L), etoposide (25 μg/mL) and PMA (100 ng/mL), and β-actin was shown as a loading control. All data are shown as the mean ± SD and the experimental points show the average of at least triplicates. All experiments were repeated at least 3 times

The activation of NF-κB was examined by dual luciferase reporter assay with HepG2 cells treated with G-Rg5 (6 μmol/L), G-Rk1 (6 μmol/L), etoposide (25 μg/mL) and PMA (100 ng/mL). G-Rg5 and G-Rk1 inhibited NF-κB activation in resting state and under treatment with etoposide and PMA, activator enhancing NF-κB activity (Fig. 3A). The similar inhibitory effect was observed to downstream genes of NF-κB like IL-6 (Fig. 3B), a classic NF-κB activating gene, and IAPs genes including X-IAP, c-IAP1, c-IAP2 and survivin (Fig. 3C–F). The protein levels of X-IAP, c-IAP1, c-IAP2 and survivin were also down-regulated by G-Rg5 and G-Rk1, in both resting and NF-κB activator-stimulated cells (Fig. 3G).

### Annexin A2 knock-down enhanced anti-cancer activity of G-Rg5 and G-Rk1 in HepG2 cells

**Figure 4:**
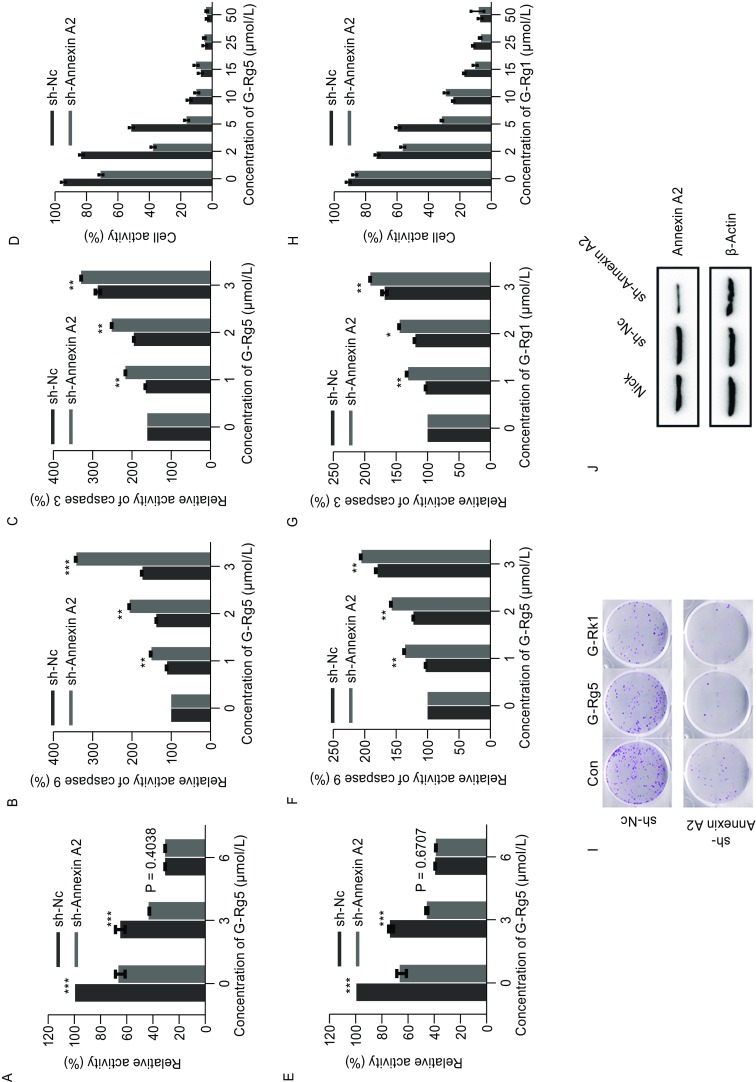
**Knock-down of Annexin A2 enhanced anti-proliferation effect of G-Rg5 and G-Rk1**. (A) NF-κB activity was examined by luciferase reporter assay under treatment of G-Rg5 with (sh-Annexin A2) or without (sh-NC) knock-down of Annexin A2. (B and C) Activity of caspase 9 (B) and 3 (C) was examined under treatment of G-Rg5 with (sh-Annexin A2) or without (sh-NC) knock-down of Annexin A2. (D) Cell viability was examined by MTT for 48 h under treatment of G-Rg5 with (sh-Annexin A2) or without (sh-NC) knock-down of Annexin A2. (E) NF-κB activity was examined by luciferase reporter assay under treatment of G-Rk1 with (sh-Annexin A2) or without (sh-NC) knock-down of Annexin A2. (F and G) Activity of caspase 9 (F) and 3 (G) was examined under treatment of G-Rk1 with (sh-Annexin A2) or without (sh-NC) knock-down of Annexin A2. (H) Cell viability was examined by MTT for 48 h under treatment of G-Rk1 with (sh-Annexin A2) or without (sh-NC) knock-down of Annexin A2. (I) Plate clone formation assay was examined under treatment of G-Rg5 (6 μmol/L) and G-Rk1 (6 μmol/L) with (sh-Annexin A2) or without (sh-NC) knock-down of Annexin A2. (J) Protein level of Annexin A2 was examined by immunoblot with (sh-Annexin A2) or without (sh-NC) knock-down of Annexin A2, and β-actin was shown as a loading control. All data are shown as the mean ± SD. *P < 0.05, **P < 0.01 and ***P < 0.001 and the experimental points show the average of at least triplicates. All experiments were repeated at least 3 times. Statistical analyses were performed using Student’s *t*-test

To examine whether Rg5 inhibits NF-κB activation primarily by Annexin A2, knock-down of Annexin A2 was employed with shRNA vectors. Both Annexin A2 knock-down and G-Rg5 inhibited NF-κB activation, and the inhibitory effect of G-Rg5 was largely enhanced by Annexin A2 knock-down at the moderate concentration (6 μg/mL), and at the high dose of G-Rg5 (6 μg/mL), Annexin A2 knock-down showed little effect (Fig. 4A), which indicates G-Rg5 regulating NF-κB mainly through Annexin A2. Caspase 3 and 9 was activated under G-Rg5 treatment, which was also enhanced by Annexin A2 knock-down (Fig. 4B and 4C). MTT assay showed the anti-survival activity of G-Rg5, which was also enhanced by Annexin A2 knock-down (Fig. 4D). G-Rk1 behaved in a similar manner to G-Rg5 on NF-κB inhibition (Fig. 4E), caspase activation (Fig. 4F and 4G) and anti-proliferation activity (Fig. 4H). A plate clone formation assay showed that G-Rg5 and G-Rk1 inhibited clone formation of HepG2 cells and Annexin A2 knock-down intensified this inhibitory effect (Fig. 4I), as expected. Taken together, G-Rg5 and G-Rk1 inhibited proliferation of HepG2 cells, which might be mainly mediated by Annexin A2.

### K302A Annexin A2 protected cells from cytotoxic effect induced by G-Rg5 and G-Rk1

**Figure 5:**
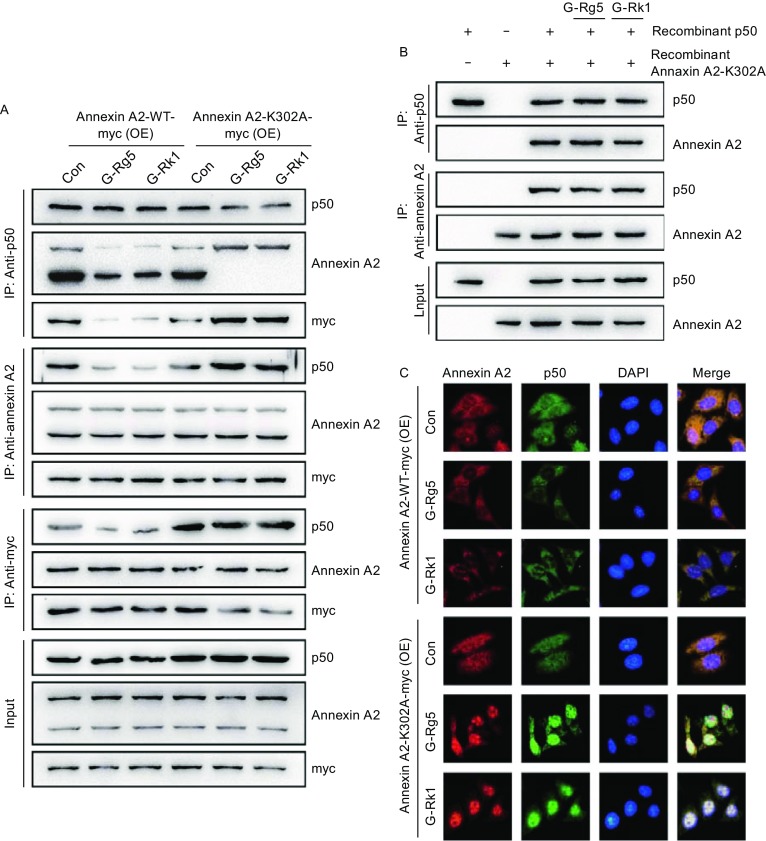
**Annexin A2-K302A blocked the inhibitory effect of G-Rg5 and G-Rk1 on the interaction between Annexin A2 and NF-κB p50 subunit and their nuclear co-localization**. (A) HepG2 cells were transfected with Annexin A2-WT-myc and Annexin A2-K302A-myc, and immunoprecipitation was performed with whole-cell lysate under treatment with G-Rg5 (6 μmol/L), G-Rk1 (6 μmol/L) and examined by an immunoblot. (B) Immunoprecipitation was performed with prokaryotic cells-expressed Annexin A2-K302A and NF-κB p50 subunit under treatment with G-Rg5 (6 μmol/L) and G-Rk1 (6 μmol/L), and the interaction was analyzed by an immunoblot. (C) HepG2 cells were transfected with Annexin A2-WT-myc and Annexin A2-K302A-myc, and the subcellular distribution of Annexin A2 and NF-κB p50 subunit was examined by immunofluorescence under treatment with G-Rg5 (6 μmol/L) and G-Rk1 (6 μmol/L), and DAPI showed the nuclear region

To further provide evidence for the major role of Annexin A2 in G-Rg5 and G-Rk1-induced NF-κB inhibition, studies were carried out with exogenous Annexin A2-K302A, a mutant version, which fails to interact with G-Rg5 and G-Rk1. First, immunoprecipitation was performed with Annexin A2-WT or Annexin A2-K302A over-expressed HepG2 cells with G-Rg5 and G-Rk1 treatment. Both chemicals attenuated the interaction between p50 and Annexin A2-WT, which was either endogenous or myc-tagged exogenous. However these two chemicals failed to reduce the interaction between p50 and Annexin A2-K302A, appeared to promote this interaction (Fig. 5A). An *in-vitro* immunoprecipitation using prokaryotic cell expressed p50 and Annexin A2-K302A showed that p50 and Annexin A2-K302A did interact with each other, and either chemicals failed to affect this interaction (Fig. 5B). Immunocytochemistry clearly showed that wild type of Annexin A2 (either endogenous or exogenous) failed to co-localize to nuclear with p50 under treatment of G-Rg5 and G-Rk1 (Fig. 5C), while the two chemicals promoted the nuclear co-localization of Annexin A2-K302A.

**Figure 6:**
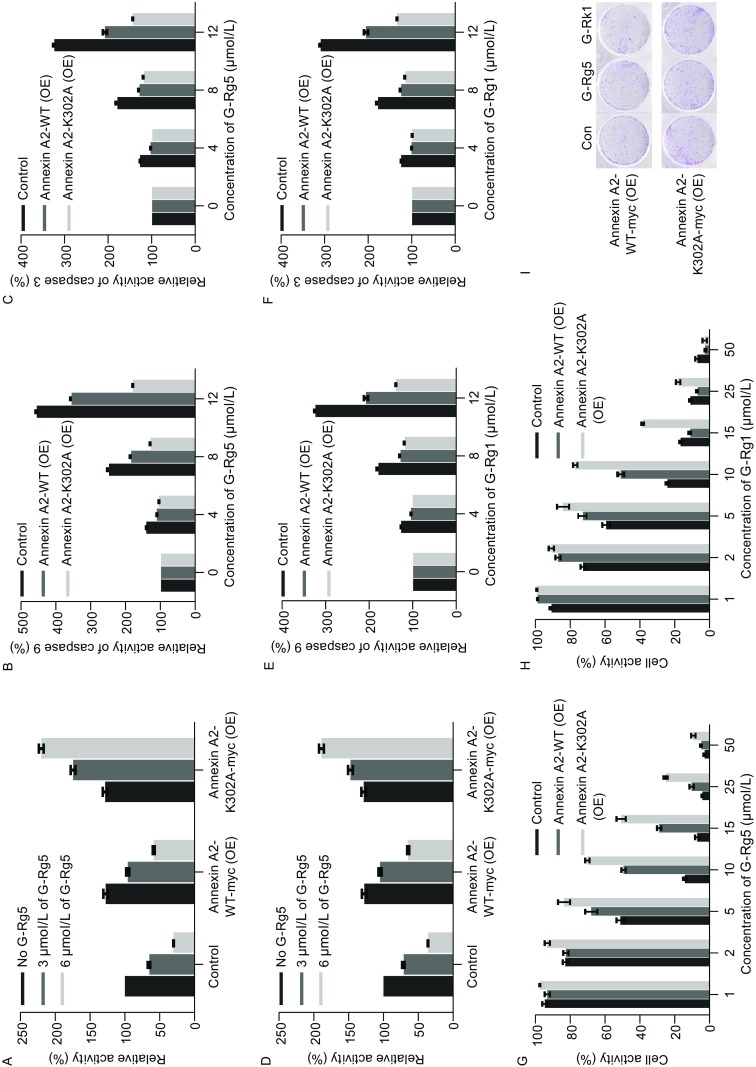
**Annexin A2-K302A protected cells from anti-proliferation effect of G-Rg5 and G-Rk1**. (A) NF-κB activity was examined by luciferase reporter assay under treatment of G-Rg5 with Annexin A2-WT-myc and Annexin A2-K302A-myc overexpressed in HepG2 cells. (B and C) Activity of caspase 9 (B) and 3 (C) was examined under treatment of G-Rg5 with Annexin A2-WT-myc and Annexin A2-K302A-myc overexpressed in HepG2 cells. (D) Cell viability was examined by MTT for 48 h under treatment of G-Rg5 with Annexin A2-WT-myc and Annexin A2-K302A-myc overexpressed in HepG2 cells. (E) NF-κB activity was examined by luciferase reporter assay under treatment of G-Rk1 with Annexin A2-WT-myc and Annexin A2-K302A-myc overexpressed in HepG2 cells. (F and G) Activity of caspase 9 (F) and 3 (G) was examined under treatment of G-Rk1 with Annexin A2-WT-myc and Annexin A2-K302A-myc overexpressed in HepG2 cells. (H) Cell viability was examined by MTT for 48 h under treatment of G-Rk1 with Annexin A2-WT-myc and Annexin A2-K302A-myc overexpressed in HepG2 cells. (I) Plate clone formation assay was examined under treatment of G-Rg5 (6 μmol/L) and G-Rk1 (6 μmol/L) with Annexin A2-WT-myc and Annexin A2-K302A-myc overexpressed in HepG2 cells. All data are shown as the mean ± SD and the experimental points show the average of at least triplicates. All experiments were repeated at least 3 times

Next, NF-κB activation was examined in Annexin A2-WT or Annexin A2-K302A overexpressed HepG2 cells. Overexpression of Annexin A2-WT enhanced NF-κB activation, and G-Rg5 inhibited NF-κB activity in a dose-dependent manner both in mock transfected and in Annexin A2-WT expressed HepG2 cells. Interestingly, G-Rg5 didn’t inhibit, even enhance NF-κB activity in Annexin A2-K302A overexpressed HepG2 cells (Fig. 6A). Activation of caspase 9 and 3 was attenuated when Annexin A2-WT was overexpressed, and only slight activation was detected with an over-expression of Annexin-K302A (Fig. 6B and 6C). Similarly, Annexin A2-K302A abolished the inhibition of NF-κB induced by G-Rk1 (Fig. 6D). G-Rk1 induced weaker caspase activation in Annexin A2-WT over-expressing cells and a little caspase activation was observed in Annexin A2-K302A over-expressing cells compared with mock transfected cells (Fig. 6E and 6F). Anti-proliferation activity was examined by MTT assay and plate clone formation assay with Annexin A2-WT or Annexin A2-K302A over-expressing HepG2 cells. Both Annexin A2-WT and Annexin A2-K302A over-expression rendered cells to resist the cytotoxic effect of G-Rg5 and G-Rk1, and the activity of Annexin A2-K302A showed higher than that of Annexin A2-WT (Fig. 6G and 6H).

## Discussion

Annexin A2, a multi-functional cancer associated protein, promotes cancer progress in a list of mechanisms. In this report we developed an assay for screening small-molecule modulator of Annexin A2 and identified two Annexin A2 regulating compounds G-Rg5 and G-Rk1 (Fig. 1A). A recent report has shown that both G-Rg5 and G-Rk1 inhibited NF-κB activation and related immuno-response in HepG2 cells (Lee, 2015). G-Rg5 and G-Rk1 directly bound to Annexin A2 and disturbed the interaction between Annexin A2 and NF-κB p50 (Figs. 1 and 2), which leaded to the cytosol-retention of these two proteins and resulted in the inactivation of NF-κB transcriptional activity. IAPs, the NF-κB downstream genes were down-regulated, and caspase-3 and -9 were activated by the treatment of either compound (Fig. 3).

As described, Annexin A2 binds to NF-κB p50 subunit, and promotes NF-κB activation, which might promote the pro-survival effect and chemoresistance in tumor tissues (Tong et al., 2015; Staquicini et al., 2017; Das et al., 2010). The N-terminal of Annexin A2 is necessary for the interaction between Annexin A2 and NF-κB p50 (Jung et al., 2015). A recent report demonstrates that Lys302 of Annexin A2 contributes to the conformational stability of its N-terminal, and promoting the interactions with other proteins driven by this sequence (Ecsédi et al., 2017). The interaction between Annexin A2 and p50 may possibly follow this pattern, and modifications towards Lys302 will probably regulate the activation of NF-κB.

G-Rg5 and G-Rk1 triggered multiple cellular events, which possibly participated in NF-κB activation, however, this activation was dominantly inactivated by G-Rg5 or G-Rk1 binding to Annexin A2-WT in Annexin A2 over-expressed cancer cells. As G-Rg5 and G-Rk1 failed to interact with Annexin A2-K302A, G-Rg5 or G-Rk1 could not inactivate the other pathway triggered NF-κB activation (Figs. 5, 6A and 6B), presenting little anti-cancer activity in Annexin A2-K302A over-expressed HepG2 cells (Fig. 6). We further examined the effect of G-Rg5 and Rk1 on NF-κB activity in various cancer cells, including SW480, PC3, HeLa and Huh7, as well as noncancerous HEK-293T cells and normal CCC-HEL1 cells. G-Rg5 or G-Rk1 significantly inhibited NF-κB activity in Annexin A2 over-expressed cells like SW480, PC3, HeLa as did in HepG2 cells, but not in Huh7, a liver cancer cell line with low expression of Annexin A2. Interestingly neither compound inhibited the NF-κB activity in noncancerous HEK-293T cell and normal CCC-HEL1 cells (Fig. S1). These data suggested that G-Rg5 or G-Rk1 may serve as a specific anticancer reagent for the treatment of Annexin A2 over-expressed cancers, and this possibility should be examined in future study.

In conclusion, we have identified two specific small-molecule inhibitors of Annexin A2, G-Rg5 and G-Rk1, which inhibited NF-κB activity and promoted apoptosis. These data supported the notion that the inhibition of Annexin A2 may be a useful strategy to prevent and treat cancers and G-Rg5 and G-Rk1 can serve as a leading compound for targeted cancer treatment.

## Materials and methods

### Cell lines, reagents and plasmids

HepG2 cells were obtained from the American Type Culture Collection (ATCC, Rockville, MA, USA). Dulbecco’s Modified Eagle Medium (DMEM) and fetal bovine serum (FBS) were obtained from Gibco BRL (Grand Island, NE, USA). Chemical reagents were listed below: G-Rg5 (Sigma), G-Rk1 (Sigma), phorbol myristate acetate (PMA, Sigma) and etoposide (Sigma). Ginsenosides were dissolved in 75% alcohol with a final concentration of 12 mmol/L. PMA (10 μg/mL) and etoposide (25 mg/mL) were dissolved in DMSO. Primary antibodies were listed below: mouse anti-Annexin A2 (Santa Cruz, sc-47696), mouse anti-p50 (Santa Cruz, sc-8414), rabbit anti-p50 (Santa Cruz, sc-7178), mouse anti-C-myc (Santa Cruz, sc-49), rabbit anti-C-myc (Santa Cruz, sc-789), mouse anti-X-IAP (Santa Cruz, sc-55551), rabbit anti-c-IAP1 (Santa Cruz, sc-7943), rabbit anti-c-IAP2 (Santa Cruz, sc-7944), mouse anti-survivin (Santa Cruz, sc-17779) and mouse anti-β-actin (Santa Cruz, sc-47778). Secondary antibodies were listed below: HRP-conjugated goat anti-mouse IgG (Pierce), HRP-conjugated goat anti-rabbit IgG (Pierce), Cy™3 affinipure donkey anti-mouse IgG (Jackson ImmunoResearch Inc., PA, USA), and Alexa Fluor® 488 affinipure donkey anti-Rabbit IgG (Jackson ImmunoResearch Inc., PA, USA). Genes of human Annexin A2 and human NF-κB p50 subunit were amplified by PCR, and cloned into pEXS-CG (GST-tag at C-terminal, provided by Professor Fei Sun’s group, at the Institute of Biophysics of Chinese Academy of Sciences). Prokaryotic vectors were gained that allowed expression of non-tagged Annexin A2 (pEXS-Annexin A2-WT), non-tagged NF-κB p50 subunit (pEXS-p50), and C-terminal GST-tagged Annexin A2 (pEXS-CG-Annexin A2-WT). A single point mutation was engineered within Annexin A2-expressing vector, with resulting vectors called pEXS-Annexin A2-K302A and pEXS-CG-Annexin A2-K302A. The sequences of both Annexin A2-WT and Annexin A2-K302A were cloned into pcs4 (C-myc-tag at C-terminal), with resulting vectors called pcs4-Annexin A2-WT-myc and pcs4-Annexin A2-K302A-myc. Short hairpin RNA (shRNA) vector, pGPU6-GFP-Neo-Annexin A2 (1496), was obtained to knock down Annexin A2 expression in RNA interference assays (GenePharma, Jiangsu, China). The luciferase reporter assays were performed with the following plasmids: pNFκB-TA-luc (Beyotime, Shanghai, China) and pRL-CMV (Promega, WI, USA).

### Molecular docking

We downloaded the three-dimensional structure of G-Rg5 (PubChem CID: 44416768) and G-Rk1 (PubChem CID: 11499198) from the NCBI Pubchem Compound database (http://www.ncbi.nlm.nih.gov/pccompound), and we downloaded the crystal structure of Annexin A2 (PDB ID: 2HYU) from the RCSB Protein Data Bank (http://www.rcsb.org/pdb). Molecular docking was performed with AutoDock tools (version 4.2.6) with the default setting, based on the Lamarckian Genetic Algorithm (Scripps Research Institute, La Jolla, CA, USA). We processed the optimum structure of the complex with the Discovery Studio 4.0 Visualizer (BIOVIA, CA, USA).

### Purification of prokaryotic-expressed Annexin A2

The *E*. *coli* expression strain, BL21 (DE3), was transformed with pEXS-CG-Annexin A2, and cultured in Luria-Bertani (LB) liquid medium with 50 μg/mL ampicillin at 37°C until the density reached an OD_600_ of 1.5. Cells were cooled to 16°C and cultured for another 12 h at 16°C with 1.0 mmol/L IPTG for protein expression. Then, cells were harvested by centrifuging at 6,000 rpm (JA10 rotor, Beckman) for 12 min. The cell pellet was resuspended in pre-cooled lysis buffer (PBS containing 1 mg/mL lysozyme, 1 mmol/L DTT and 1 mmol/L PMSF), placed on ice, and ultra-sonicated for cell lysis. The lysed cells were separated with centrifugation at 12,000 rpm (JA25.50 rotor, Beckman) for 40 min, and the supernatant was loaded onto a GST-affinity chromatography column. The column was washed with pre-cooled PBS containing 1 mol/L NaCl, 1 mmol/L DTT and 1 mmol/L PMSF. Then, we added 10 μg of human HRV 3C protease (TAKARA) to the column, and incubated the column at 4°C overnight to allow cleavage of the GST-tag at the C-terminus of the protein. The eluted fraction was then loaded onto a Superdex75 16/600 column (GE Healthcare) and eluted at a flow rate of 1.0 mL/min. Fractions were pooled and concentrated to 10 mg/mL with a 10-kDa cut-off centrifuge filter (Millipore).

The Annexin A2-K302A mutant was purified with the same procedure.

### Thermal shift assay

*In vivo* thermal shift assay (cellular thermal shift assay, CTSA): HepG2 cells (3 × 10^7^) were seeded into a 100-mm culture plate with 12 μmol/L of G-Rg5 or G-Rk1 and cultured for 1 h. Control cells were incubated with the same volume. Cells were cultivated and counted, followed by resuspending in PBS (containing 1 mmol/L PMSF) to a final density of 2 × 10^7^/mL. Then cells were subpackaged into 12 PCR tubes, with 100 μL per PCR tube, and heated with a thermal gradient from 40°C to 73°C, for 3 min. After freeze-thawed twice with liquid nitrogen, the supernatant was separated by centrifugation at 20,000 ×*g* for 20 min and collected. 20 μL of the supernatant was loaded onto an SDS-PAGE gel, followed by an immunoblot.

*In vitro* thermal shift assay: Purified Annexin A2 protein was diluted with PBS (containing 1 mmol/L PMSF) to a final concentration of 0.2 mmol/L (~7 μg/mL) and subpackaged into PCR tubes, with 90 μL per PCR tube. G-Rg5 or G-Rk1 were diluted with PBS to a final concentration of 100 μmol/L, then added to PCR tubes with Annexin A2 protein, forming a dose gradient from 0.2 μmol/L to 10 μmol/L. PCR tubes were made up to 100 μL with PBS and heated at 55°C for 3 min, with the control tube on ice for 3 min. Then 10 μL from each tube was loaded onto an SDS-PAGE gel, followed by an immunoblot.

### Immunoprecipitation

*In vivo*: HepG2 cells (1.6 × 10^6^) were plated into 100-mm culture plates and treated with G-Rg5 (6 μmol/L), G-Rk1 (6 μmol/L), PMA (100 ng/mL) and etoposide (25 μg/mL) for 12 h. Cells were lysed in lysis buffer (Pierce) containing protease inhibitors (Roche) and 1 mmol/L PMSF. 2 mg of total protein lysates were mixed with 10 μL of anti-Annexin A2, anti-p50 antibody or anti-C-myc, and incubated at 4°C for 3 h on a tube rotator. Protein A/G beads (Millipore) were washed three times with lysis buffer, and then incubated at 4°C for 4 h with the lysate-antibody complexes. The protein-agarose beads complexes were washed three times with lysis buffer. Samples were then separated with an SDS-PAGE and analyzed with an immunoblot.

*In vitro*: Cultures of the *E*. *coli* expression strain, BL21 (DE3), were transformed with pEXS-CG, pEXS-Annexin A2 (label-free) and pEXS-p50. Cells were cultivated in LB liquid medium containing 50 μg/mL ampicillin at 37°C, until the cell density reached an OD_600_ of 1.5. Then, cells were cultivated with 1 mmol/L IPTG for another 12 h. Cells were lysed with ultra-sonication in lysis buffer (100 mmol/L PBS, 125 mmol/L NaCl, 1 mg/mL lysozyme, and 1 mmol/L PMSF). 1 mg of the protein lysate containing Annexin A2 and another 1 mg of the protein lysate containing p50 were incubated at 4°C overnight with 12 μmol/L G-Rg5 or G-Rk1. As a control, 1 mg of protein lysate that contained neither Annexin A2 nor p50 was incubated in parallel. The remaining steps were performed as described above for the *in vivo* assay.

### Immunofluorescence

Glass cover slips were placed into the wells of a 24-well plate, and 5 × 10^4^ HepG2 cells were seeded into each well. After a 16-h incubation, cells were treated with G-Rg5 (6 μmol/L), G-Rk1 (6 μmol/L), PMA (100 ng/mL) and etoposide (25 μg/mL) for 2 h. Next, cells were fixed with pre-cool methanol at 4°C for 5 min. Then, cells were washed twice with PBST (100 mmol/L PBS with 0.5% Tween-20). The fixed cells were permeabilized with PBST containing 0.2% Triton X-100 at 4°C for 20 min, then washed three times with PBST. Permeabilized cells were incubated with blocking buffer (PBST containing 3% donkey serum) at room temperature for 1 h, then incubated with a primary antibody (mouse anti-Annexin A2 and rabbit anti-NF-κB, each diluted 1:200 in PBST with 5% BSA) for 3 h at 4°C. Cells were washed three times with PBST and incubated with a secondary antibody (Cy™3 affinipure donkey anti-mouse IgG and Alexa Fluor® 488 affinipure donkey anti-Rabbit IgG, each diluted 1:200 in PBST with 5% BSA) for 2 h at room temperature. After the cells were washed twice with PBST, stained with PBST and 0.1% DAPI (Sigma), and analyzed with a fluorescence microscope.

### Cell viability assay

HepG2 cells were plated (5 × 10^3^ per well) onto 96-well plates and treated with G-Rg5 or G-Rk1 at the indicated concentrations for 48 h. Cell viability was determined with the MTT assay.

### Plate clone formation assay

HepG2 were plated (1 × 10^3^ per well) onto 6-well plates and treated with G-Rg5 (6 μmol/L) and G-Rk1 (6 μmol/L) at the indicated concentrations for 1 week. Then cells were washed twice with PBS and fixed with pre-cooled methanol for 10 min, followed by crystal violet staining for 5 min. Then cells were washed twice with PBS and photographed.

### Quantitative real-time PCR (qRT-PCR)

Whole-cell RNA was isolated with TRIzol (Invitrogen). Then, 5-μg aliquots of whole-cell RNA were used for cDNA synthesis with the EasyScript Reverse Transcriptase kit (Transgen). QRT-PCR was performed on Applied Biosystem 7500 Real-time PCR system (Applied Biosystem. Inc) and TransStart Tuo Green qPCR SuperMix (Transgen). The amplification was performed with a three-step program, 1 cycle at 94°C for 30 s, followed by 45 cycles of 94°C for 5 s, 50°C for 15 s, and 72°C for 10 s, with signal collecting steps after annealing and extending for 34 s.

### Statistical analysis

Data are presented as the mean ± S.D. Statistical significance was calculated with the Student’s *t*-test.

## Electronic supplementary material

Below is the link to the electronic supplementary material.

Supplementary material 1 (PDF 503 kb)
