# Supplementary material for: Identification of natural compounds targeting Annexin A2 with an anti-cancer effect
Source: Protein Cell. 2018 Mar 5;9(6):568–79. doi: 10.1007/s13238-018-0513-z (PMC5966357; doi:10.1007/s13238-018-0513-z)
Supplement: Supplementary file 1 — Supplementary material 1 (PDF 503 kb) [file 13238_2018_513_MOESM1_ESM.pdf]

Figure S1. G-Rg5 and G-Rk1 inhibited NF-κB in an Annexin A2-dependent manner in cancer cell lines but normal cells. (A) NF-κB activation was examined various cell lines, including HepG2, SW480, PC3, Huh7, HeLa, HEK-293T and CCC-HEL1. (B) Protein level of Annexin A2 in various cell lines was examined by immunoblot and β-actin functioned as a loading control.

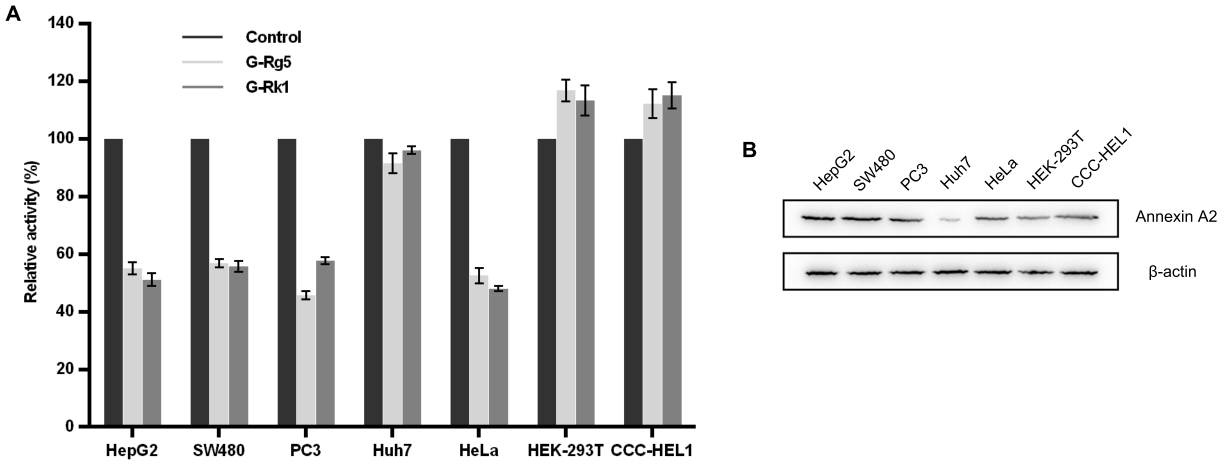
A
Control
G-Rg5
G-Rk1
Relative activity (%)
140
120
100
80
60
40
20
0
HepG2
SW480
PC3
Huh7
HeLa
HEK-293T
CCC-HEL1
B
HepG2
SW480
PC3
Huh7
HeLa
HEK-293T
CCC-HEL1
Annexin A2
β-actin
